# Prevalence of depression and associated factors among HIV/AIDS patients attending antiretroviral therapy clinic at Adama Hospital Medical College, Adama, Central Ethiopia

**DOI:** 10.1038/s41598-024-52142-z

**Published:** 2024-01-18

**Authors:** Tessema Gebru, Daba Ejara, Aster Yalew, Negussie Deyessa

**Affiliations:** 1 HIV Directorate, Oromia Regional Health Bureau, Addis Ababa, Ethiopia; 2 Department of Nursing, Shashamene Campus, Madda Walabu University, Shashamene, Ethiopia; 3 School of Public Health, College Health Science, Addis Ababa University, Addis Ababa, Ethiopia

**Keywords:** Neuroscience, Health care, Medical research, Neurology

## Abstract

Depression is the most frequently detected and preventable mental illness among people with human immunodeficiency syndrome, with rates two to four times higher than in the general population. Currently, depression is estimated to affect 350 million people worldwide. To assess the prevalence of depression and associated factors among HIV/AIDS patients attending antiretroviral therapy clinic at Adama Hospital Medical College, Adama, Central Ethiopia. An institutional-based cross-sectional study was conducted from April 01 to September 30, 2021, at Adama Hospital Medical College, Adama, Ethiopia. A total of 420 individuals were selected using a systematic random sampling technique. After informed consent was obtained from each study participant, data were collected through face-to-face interviews, observations, and document reviews. Subsequently, the data were entered into EPI-Info Version 7 and analyzed by Statistical Package for the Social Sciences version 21. Variables with p-values less than 0.25 in the univariable logistic regression analysis were subsequently included in the multivariable logistic regression analysis to account for potential confounding factors. The association was measured using adjusted odds ratio (AOR) with a 95% confidence interval (CI), and variables with *p*-values less than 0.05 were considered statistically significant. The prevalence of depression was 52.4% (95% CI 47.6–57.1). Factors significantly associated with depression among HIV-positive patients on antiretroviral therapy included employment status [AOR = 0.22(95% CI 0.13–0.36)], the patient’s most CD4 count [AOR = 6.99 (95% CI 2.81–17.38)], duration of months on antiretroviral therapy [AOR = 5.05 (95% CI 2.38–10.74)] and presence of chronic non-communicable diseases [AOR = 7.90 (95% CI 4.21–14.85)]. The highest proportion of HIV-positive patients taking antiretroviral drugs exhibited depression. Employment was identified as a preventive factor, whereas having a low CD4 count, recently initiating antiretroviral therapy, and having chronic non-communicable diseases were associated with increased odds of depression among HIV-positive patients on antiretroviral therapy. There need to strengthen mental health screening and treat depression among HIV-positive patients, particularly by targeting identified factors.

## Introduction

Depression is one of the psychiatric disorders among people living with human immunodeficiency virus/acquired immunodeficiency syndrome (HIV/AIDS) and manifests as loss of interest, depressed mood, changes in sleep, change in appetite, poor psychomotor activity, difficulty in making a decision, uncomfortable or immoral feelings, and quickly getting fatigued and constant feelings of death or suicide^1^. According to World Health Organization (WHO), 1 in every 8 individuals worldwide live with mental health disorder in 2022. In the same year, 280 million people were living with depression^2^. Depression is the most common and easily treatable mental disorder in patients with human immunodeficiency syndrome, with rates two to three times higher than in the general population^3,4^. Prior studies have reported that people living with HIV/AIDS (PLWHA) are at a significantly higher risk of neuropsychiatric comorbidities, with depression being the most common (20–40%)^5^. Studies conducted in different countries showed that the prevalence of depression among HIV/AIDs patients was 57% in India^6^, 40.9% in China^7^ and 32.2% in Pakistan^8^. This is often related to the fact that PLWHA frequently endure social stigma, loss of social support, loneliness, and low self-esteem. Furthermore, a higher chronicity of depression has also been linked to HIV appointment attendance, treatment failure, and mortality among HIV-infected people^5,9^.

Studies have found a high rate of depression among HIV-positive patients in Sub-Saharan Africa, which is home to 67% of the world's HIV-positive patients^10^. According to a systematic review and meta-analysis in the East African region, the prevalence of depression among PLWHA was 38%^11^. Similarly, studies have shown the prevalence of depression was 63.1% in Sudan^12^, 46% in Western Uganda^13^, 33% in Somalia^14^, and 26.7% in Cameroon^15^. This high prevalence of depression among HIV/AIDS individuals is associated with reduced treatment adherence, which can increase disease progression and mortality in high, middle and low-income countries^16–18^.

In Ethiopia, the prevalence of depression among people living with HIV/AIDS was 48.6% in Hawassa^19^, 44.9% in Southeast Ethiopia^20^, 45.8% in Harar^21^, 41.7% in Gimbi^22^, 38.9% in Debrebirehan^23^, 35.5% in Addis Ababa^24^, 30.2% in Jimma^25^ and 11.7% in Debre Markos^26^.

Mental health problem related to HIV infection is quite recurrent due to stressful events such as the emotional impact of diagnosis, possible family rejection in professional and social life; stigmatization and discrimination associated with the disease and clinical features, chronic course of the disease, and side-effects of certain antiretroviral medications such as Zidovudine and other. Studies have shown that urban dwellers, lower socioeconomic class, unemployed and government employees, female sex, history of hospital admission, discontinued education due to HIV/AIDS illness, poor treatment adherence, opportunistic infections, high baseline viral load, and 6 months duration of HIV diagnosis were factors associated with depression among HIV positive patients. Hence, depression among PLWHA taking antiretiroviral therapy (ART) is still underdiagnosed and under-treated; there is a need to incorporate mental health services as an integral component of HIV care^17,21,25,27^.

Depression has been linked to a variety of negative health outcomes in HIV/AIDS patients, including suicidal attempts, hopelessness, and poor drug adherence leading to rapid HIV progression, which in turn will result in drug resistance and treatment failure. Unless promptly recognized and managed, depression and its negative consequences will result in hospitalization and an increase in the cost of medical care^17,27^.

Although there have been studies on the prevalence and associated factors of depression among HIV-positive patients in Ethiopia, none has been done at Adama Hospital Medical College, which has over 7000 HIV-positive patients taking ART drugs. Most importantly, due to variation among the study populations both within and between countries, the prevalence and associated factors of depression may, sometimes, be population specific. Therefore, this study was done to assess the burden of depression among HIV-positive patients in the study area, generate evidence for early recognition, and strengthen the integration of mental health care into ART services.

This study aimed to investigate the prevalence and associated factors of depression among HIV-positive patients attending ART clinic at Adama Hospital Medical College, Adama, Ethiopia.

## Methods and materials

### Study area and period

The study was conducted from April 01 to September 30, 2021, at Adama Hospital Medical College in Adama town, Eastern Oromia Region, Ethiopia. Adama is found 100 km away from Addis Ababa in the East path. At different times, Adama Hospital Medical College was identified by the names of Haile Mariam Mamo Memorial Hospital and Adama Referral Public Hospital. It is the only public Medical Hospital situated in Adama town.

The hospital was established in 1946 GC by missionaries from abroad and was among the country’s first non-governmental hospitals. It was handovered by the government during the Dergue regime in 1974. The hospital was upgraded to a Medical College in 2011 G.C and started to play academic and research roles in addition to its normal medical service, development, and administrative activities. This hospital and college currently serve over six million catchment population from five regions (Oromia, Amhara, Afar, Somali, and Dire-Dawa). Regarding the HIV/AIDS services, there are different professional categories assigned like General Practitioners, Heath officers, Nurses, Case managers, Mother support groups, Pharmacy technologists and others who took training on this specific program. There are more than 7000 patients currently taking ART drugs in this hospital, and they are usually served by follow-up on the Appointment Spacing model, three multi-month dispensaries and monthly basis.

### Study design and population

An institutional-based cross-sectional study design was employed. The source population was all adult HIV-positive people visiting the Adama Hospital Medical College ART clinic during the study period. The study population was all selected adults aged ≥ 18 years at the time of study and critically ill ART users, and those who could not respond appropriately to the interview were excluded from the study.

### Sample size and sampling procedure

The sample size was computed using Epi Info 7 stat calc. Using a population survey formula proportion for a single population, assuming a level of confidence at 95% and margin of error at 5%, and based on a study done at Harari town, revealed a prevalence of 45.8% of depression^9^. A 10% non-response rate was added to obtain a maximum sample size, and the final sample size was 420. Systematic random sampling was used to select participants using the antiretroviral drug registration book as the sampling frame. An interval of k = N/n = 7000/420 = 16 was used to select the study participants. The first study participant was selected using the lottery method and then every 16th client was included. Study participants were invited to participate when attending the hospital for antiretroviral drug collection.

### Data collection procedure and tools

Data were collected using an interviewer-administered questionnaire that covered socio-demographic, psychological, and social characteristics, the presence of chronic non-communicable diseases, health and nutrition-related conditions, and Patient Health Questionnaire (PHQ-9). Depression was assessed using PHQ-9 quick depression assessment, which ranges from 0 to 27. The severity of depression was characterized as minimal (0–4), mild (5–9), moderate (10–14), moderately severe^15–19^ and severe depression (≥ 20)^28^. The questionnaire was adapted from different kinds of literature^17,21,28^ and was pre-tested on 5% of the study population in the non-selected hospital /Bishoftu Hospital/ to ensure clarity, wording, logical sequence, and skip patterns of the questions. The questionnaire was prepared in English and then translated into local language (Afaan Oromo) and Amharic by language experts. To ensure consistency, the Afaan Oromo version was translated back into English. Data was collected using both an Afaan Oromo and Amharic language questionnaire based on the patient’s preferences.

Four BSc nurses were recruited as data collectors, and one master’s degree holder was assigned as the supervisor. The training was given to data collectors and supervisor for one day on data collection methods, how to take informed consent, how to approach participants, ethical procedures, and general information on the depression grade of HIV patients taking ART, and the study’s objective. Face-to-face interview, observation and document review was done to collect the data. Document review were used to fill in information like CD4 count, Viral load, and WHO clinical staging. The collected data underwent daily checks for activity, consistency, and questionnaire completeness to ensure data quality. Incomplete or unfilled questionnaires were not accepted.

### Data analysis methods

After data collection, a questionnaire was checked for missed data and errors, and then data was entered into Epi-info version 7 and analyzed using the statistical package for Social Science (SPSS) version 21. The data were cleaned and prepared for analysis. Descriptive statistics such as frequency, mean, and standard deviation describe the study’s variables. A binary logistic regression model was used to ascertain the relationship between the independent variables and the outcome variable. Assumptions for logistic regression were considered, and model fitness was tested by Hosmer and Lemeshow goodness-of-fit test statistic and was born at greater than 0.05. Before including factors, multicollinearity was checked using the cutoff point, variance inflation factor (VIF) < 10. Factors with a p-value less than 0.25 in the univariable logistic regression analysis were further entered into the multivariable analysis to control for potential confounders. Adjusted Odds Ratio with 95% CI was used to measure association, and p-values less than 0.05 were taken as statistically significant. Finally, the results of the findings were presented using text, graphs and tables.

### Ethical considerations

The study was approved by Addis Ababa University institutional review board, and a formal letter of permission was obtained from Addis Ababa University, the school of public health with registration number SHP/0023/13. An official letter of cooperation was written from Addis Ababa University to Oromia Regional Health Bureau, which then wrote to the respective health facility. There is no potential risk that may cause any harm to respondents. All the necessary precaution for COVID-19 prevention was kept during data collection. The importance of the study was explained to the study participants, and informed written consent was requested from the subjects included in the survey immediately before the data collection with the subjects full right to refuse the interview at any time if they did not want to proceed. The respondents were informed that they would not lose anything (do not affect their treatment regimen) for not participating in the study. Patients who were diagnosed with depression during data collection were linked to a psychiatry unit for further evaluation and treatment.

### Operational definition

*Depression*:—a person who scores in the PHQ-9 greater or equal to the cutoff point of 5 and has a common mental illness that negatively affects how they feel, the way they think and how they act^28^.

*Good adhered to ART drugs*:—patients who take ≥ 95% of the prescribed dose were considered adherent to medication as stated in Ethiopian consolidated ART guideline^29^.

*Mody Mass Index (MBI)*: underweight if the patient had a BMI < 18.5 kg/m^2^, normal weight if the patient had a BMI between 18.5 and 24.9 kg/m^2^ and overweight if the patient had a BMI > 25.0 kg/m^2^^30^.

### Ethical approval and consent to participate

The study received ethical approval from the AAU Ethical Review Committee and written informed consent from each participant. All methods were carried out in accordance with relevant guidelines and regulations.

## Result

### Socio-demographic characteristics of study participants

Four hundred forty-two HIV-positive patients participated in the study, and the response rate was 100%. The patients’ mean (± standard deviation) age was 42.8(± 10.7) years. More than half, 64.0% (n = 269) of the patients were females. The majority (88.1%) (n = 370) were urban residents. Nearly one-third, 36.4% (n = 153) of the study participants attended primary school. About half, 48.1% (n = 202) of the participants were married. Among the study subjects, 46.9% (n = 197) of PLWHA were unemployed. Nearly half of the participants, 51.9% (n = 218), had an average monthly income of 1001–5000 Ethiopian Birr (Table 1).

**Table 1:** Socio-demographic characteristics of patients taking ART in Adama Hospital Medical College, 2021.

Variables (n = 420)	Category	Frequency (%)
Age of the patients (Years)	18–24	25 (6.0)
	25–34	49 (11.7)
	35–44	162 (38.6)
	≥ 45	184 (43.8)
Sex of the patients	Male	151 (36.0)
	Female	269 (64.0)
Residence	Urban	370 (88.1)
	Rural	50 (11.9)
Educational status of the patients	No formal education	88 (21.0)
	Primary education	153 (36.4)
	Secondary education	123 (29.3)
	Tertiary education	56 (13.3)
Marital status	Married	202 (48.1)
	Divorce/separated	77 (18.3)
	Single	47 (11.2)
	Widowed	87 (20.7)
	Separate	7 (1.7)
Work status	Employed	223 (53.1)
	Unemployed	197 (46.9)
Income (ETB)	< 4100	384 (91.4)
	4101–6500	25 (6.0)
	≥ 6501	11 (2.6)

### Psychological and social characteristics of HIV-positive patients

Only (10%) (n = 42) and 2.9% (n = 9) of the study participants consumed alcohol and chew chat, respectively. Almost all 99.5% (n = 418) patients were non-smokers. Twenty-five (6.0%) of the study participants had a past psychiatric history. Nearly one-fifth (19%) (n = 90) of HIV-positive patients have co-morbid psychiatric conditions, while 2.9% (n = 12) were taking antidepressants. Among the study participants, 7.6% (n = 32) replied that they had poor social support. The majority, 86.4% (n = 363), had no impairments in activities of daily living (Table 2).

**Table 2:** Psychological and social characteristics of patients taking ART in Adama Hospital Medical College, 2021.

Variables (n = 420)	Category	Frequency (%)
Alcohol consumption	Yes	42 (10.0)
	No	378 (90.0)
Chewing chat	Yes	12 (2.9)
	No	408 (97.1)
Smoking cigarettes	Yes	2 (0.5)
	No	418 (99.5
Past psychiatric history	Yes	25 (6.0)
	No	395 (94.0)
Co-morbid psychiatric condition	Yes	80 (19.0)
	No	340 (81.0)
Taking antidepressants	Yes	12 (2.9)
	No	408 (97.1)
Family history of psychiatric illness	Yes	25 (6.0)
	No	395 (94.0)
Poor social support	Yes	32 (7.6)
	No	388 (92.4)
Living companion	Yes	357 (85.0)
	No	63 (15.0)
Sources of financial support	Self	307 (73.1)
	Other sources	113 (26.9)
Impairment in activities of daily living	Yes	57 (13.6)
	No	363 (86.4)

### Chronic non-communicable diseases among PLWHA

Among HIV-positive patients, 8.6% (n = 36) had diabetes mellitus. Thirty-three participants (7.9%) had hypertension, and 2.6% (n = 11) had cardiac problems. One in ten, 10.5% (n = 44) of the study participants had a history of Asthma attacks. 27.9% (n = 117) of the survey participants had at least one chronic non-communicable disease (Table 3).

**Table 3:** Presence of chronic Non-communicable diseases among patients taking ART in Adama hospital medical college, 2021.

Variables (n = 420)	Category	Frequency (%)
Diabetes	Yes	36 (8.6)
	No	384 (91.4)
Hypertension	Yes	33 (7.9)
	No	387 (92.1)
Cardiac diseases	Yes	11 (2.6)
	No	409 (97.4)
Asthma	Yes	44 (10.5)
	No	376 (89.5)
Chronic non-communicable diseases	Yes	117 (27.9)
	No	303 (72.1)

### Health and nutrition related conditions of HIV-positive patients

Nearly half, 51.7% (n = 217) of the study participants were concordant positive and one-third, 33.3% (n = 140) of the HIV-positive patients don’t know their partner’s HIV status. Regarding viral suppression, 95.5% (n = 401) of the participants had a viral load of < 1000 copies/mm^3^. Nearly two-thirds, 63.8% (n = 268) of the patients presented with CD4 < 200 cells/mm^3^ at diagnosis. Regarding the most recent CD4, 37.9% (n = 159) of the study participants had ≥ 500 cells/mm^3^. The majority, 79.5% (n = 334) of the HIV-positive patients, were in the stage 1 WHO clinical stage. Nearly one-third, 36.9% (n = 155)of HIV-positive patients had ever developed an opportunistic infection. The majority, 91.7% (n = 385) and 72.4%(n = 304) of the participants were on first-line treatment and had normal BMI, respectively (Table 4).

**Table 4:** Health and Nutrition related conditions of patients taking ART in Adama Hospital Medical College, 2021.

Variables (n = 420)	Category	Frequency(%)
Partner HIV status	Positive	217 (51.7)
	Negative	63 (15.0)
	Unknown	140 (33.3)
Viral load	≥ 1000 copies/mm^3^	19 (4.5)
	< 1000 copies/mm^3^	401 (95.5)
CD4 at diagnosis	< 200 cells/mm^3^	268 (63.8)
	200–499 cells/mm^3^	126 (30.0)
	≥ 500 cells/mm^3^	26 (6.2)
Most recent CD4 count	< 200 cells/mm^3^	67 (16.0)
	200–499 cells/mm^3^	194 (46.1)
	≥ 500 cells/mm^3^	159 (37.9)
WHO clinical stage	Stage 1	334 (79.5)
	Stage 2	41 (9.8)
	Stage 3	33 (7.8)
	Stage 4	12 (2.9)
Ever developed opportunistic infection	Yes	155 (36.9)
	No	265 (63.1)
Treatment level	First line	385 (91.7)
	Second line	35 (8.3)
ART dose is given per day	Once daily	371 (88.3)
	Twice daily	48 (10.2)
	Three times daily	6 (1.4)
Months on ART	≤ 24 months	76 (18.1)
	> 24 months	344 (81.9)
Any side effects of ART medication	Yes	44 (10.5)
	No	376 (89.5)
Currently taking any treatment other than ART	Yes	41 (9.8)
	No	379 (90.2)
Adherence to ART medication	Adhered	400 (95.2)
	Not adhered	20 (4.8)
HIV disclosure status	Disclosed	310 (73.8)
	Not disclosed	110 (26.2)
Felt stigmatization	Yes	132 (31.4)
	No	228 (68.6)
Daily eating pattern	Three meals or more	368 (87.6)
	Two meals or less	52 (12.4)
BMI	Underweight	45 (10.7)
	Normal	304 (72.4)
	Overweight	71 (16.9)

### Depression among HIV Positive patients

**Figure 1:**
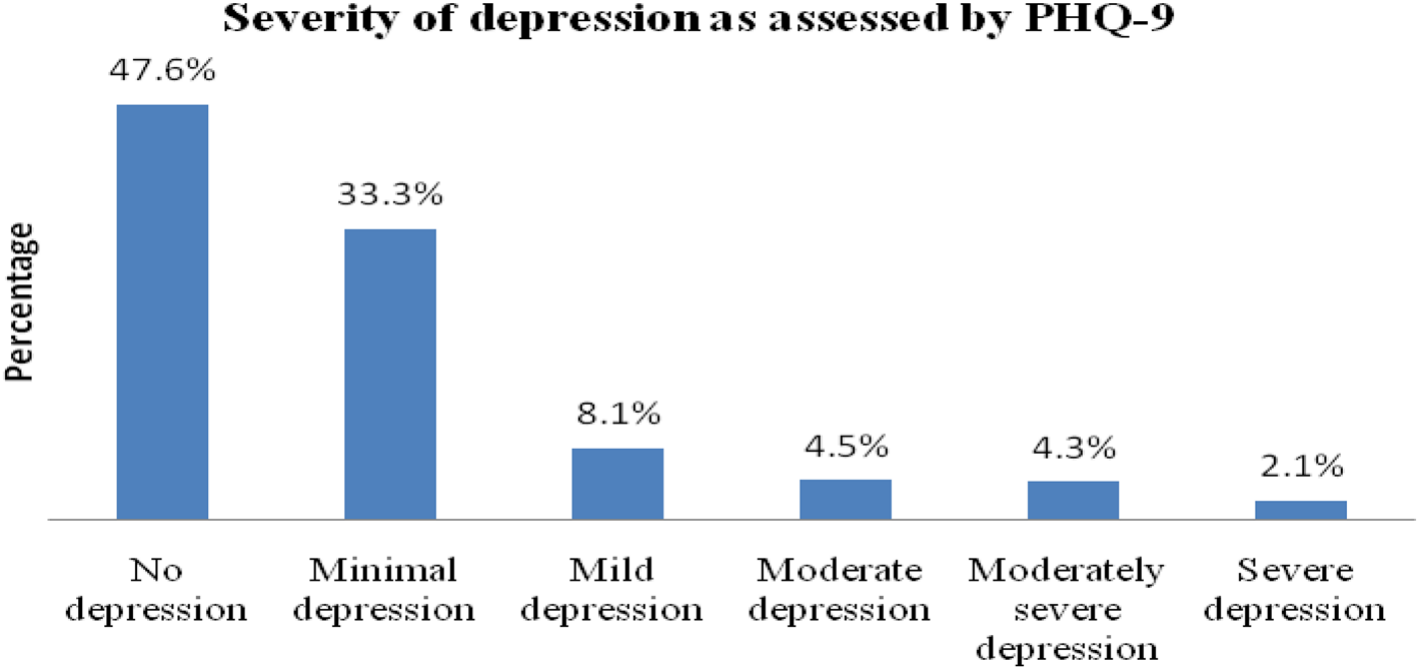
Severity of depression among HIV-positive patients taking ART at Adama Hospital Medical College, 2021.

Based on the PHQ-9 depression severity assessment scale, 33.3% (n = 140) of the study participants have minimal depression, while 9 (2.1%) have severe depression (Fig. 1).

**Figure 2:**
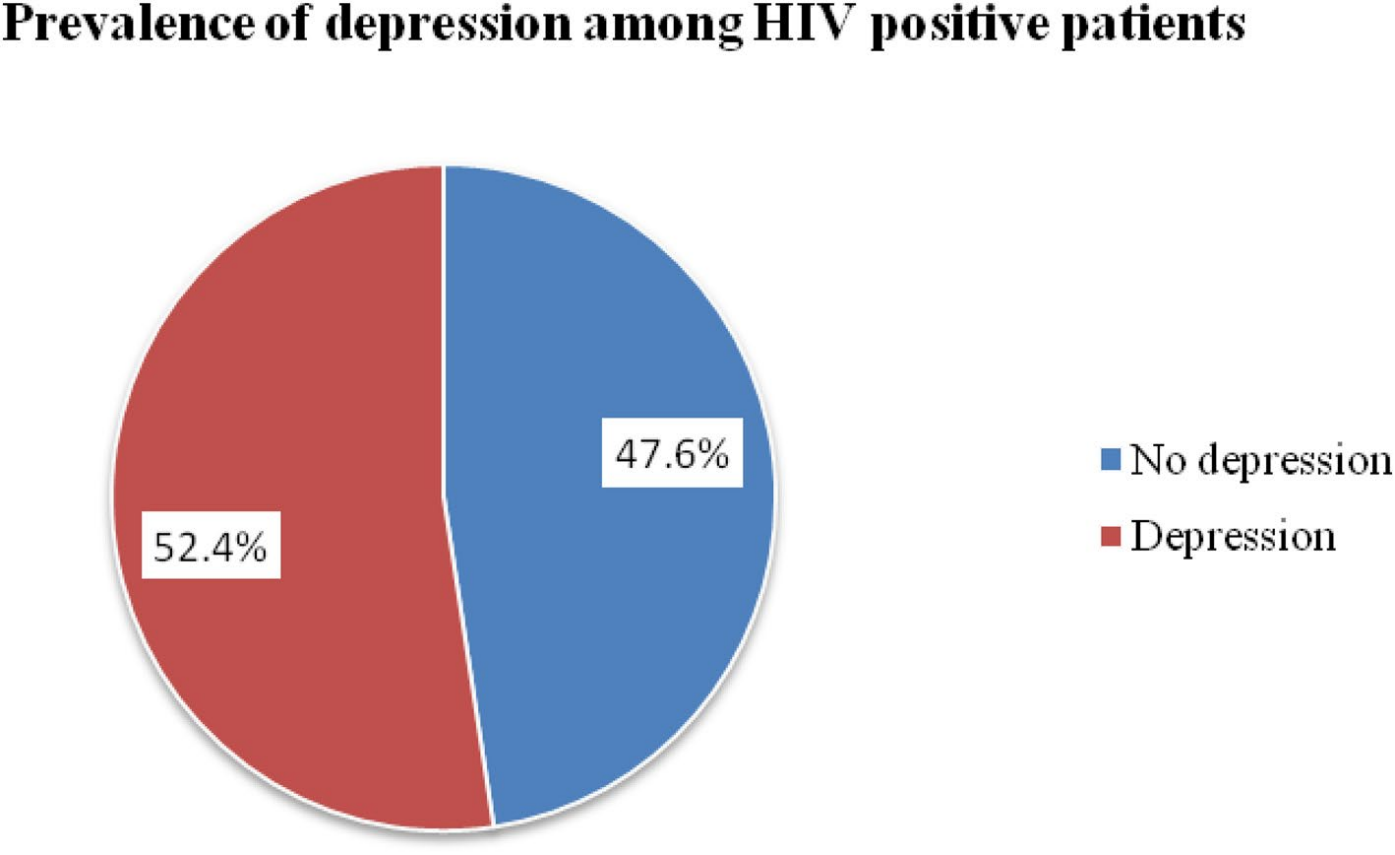
Prevalence of depression among HIV-positive patients taking ART at Adama Hospital Medical College, 2021.

The prevalence of depression among patients taking ART in Adama Hospital Medical College was 52.4% (95% CI 47.6–57.1) (Fig. 2).

### Factors associated with depression among HIV-positive patients

The univariable logistic regression analysis revealed that depression among HIV-positive patients had an association with the educational level of the patients, work status, chewing chat, family history of psychiatric illness, patient’s most recent CD4 count, months on ART and presence of chronic non-communicable diseases. Those variables with a p-value of less than 0.25 in the simple logistic regression analysis were entered in multivariable logistic regression analysis. In multivariable logistic regression analysis, work status, patient’s most recent CD4 count, months on ART and chronic non-communicable diseases were significantly associated with depressive symptoms among HIV-positive patients.

The odds of employed patients having depressive symptoms were 78% [AOR = 0.22(95% CI 0.13–0.36)] lesser than unemployed patients. HIV-positive patients who had the most recent CD4 count < 200 cells/mm^3^ were seven times [AOR = 6.99 (95%CI 2.81–17.38)] more likely to have depression than patients with CD4 count ≥ 500 cells/mm^3^. Patients who received ART for ≤ 24 months were five times [AOR = 5.05 (2.38–10.74)] more likely to have depression than their counterparts. HIV-positive patients with co-morbid chronic non-communicable disease were eight times [AOR = 7.90 (4.21–14.85)] more likely to have depression than their counterparts (Table 5).

**Table 5:** Determinants of depression among HIV-positive patients in Adama Hospital Medical College, 2021.

Variables (n = 420)	Category	Depression		COR (95% CI)	AOR (95% CI)
		Yes n = 220 (%)	No n = 200 (%)		
Level of education	No formal education	50 (56.8)	38 (43.2)	2.19 (1.10–4.35)	1.45 (0.59–3.54)
	Primary education	84 (54.9)	69 (45.1)	2.02 (1.08–3.80)	1.83 (0.81–4.14)
	Secondary education	65 (52.8)	58 (47.2)	1.87 (0.98–3.56)	1.46 (0.63–3.36)
	Tertiary education	21 (37.5)	35 (62.5)	1	1
Work status	Employed	84 (37.7)	139 (62.3)	0.27 (0.18–0.41)	0.22 (0.13–0.36)**
	Unemployed	136 (69.0)	61 (31.0)	1	1
Chat chewing	Yes	11 (91.7)	1 (8.3)	10.47 (1.34–81.87)	6.82 (0.69–67.23)
	No	209 (51.2)	199 (48.8)	1	1
Family history of psychiatric illness	Yes	20 (80.0)	5 (20.0)	3.90 (1.43–10.59)	3.04 (0.92–10.06)
	No	200 (50.6)	195 (49.4)	1	1
Most recent CD4 count	< 200 cells/mm^3^	59 (88.1)	8 (11.9)	9.14 (4.09–20.38)	6.99 (2.81–17.38)*
	200–499 cells/mm^3^	90 (46.4)	104 (53.6)	1.10 (0.70–1.63)	1.03 (0.62–1.71)
	≥ 500 cells/mm^3^	71 (44.7)	88 (55.3)	1	1
Months on ART	≤ 24 months	64 (84.2)	12 (15.8)	6.43 (3.35–12.34)	5.05 (2.38–10.74)**
	> 24 months	156 (45.3)	188 (54.7)	1	1
Chronic non-communicable diseases	Yes	99 (84.6)	18 (15.4)	8.27 (4.76–14.37)	7.90 (4.21–14.85)**
	No	121 (39.9)	182 (60.1)	1	1

NB: 1- Reference group, *P < 0.05, **P < 0.0.

## Discussion

This study set out to examine the prevalence and factors associated with depression among patients taking antiretroviral drugs in the study setting. We found that 52.4% of HIV-positive patients taking ART had depression. This finding is comparable with a study conducted in India (57%)^6^, Hawassa (55.8%)^19^, Western Uganda (46%)^13^, and Harar town (45.8%)^21^. This finding is higher than a study done in China (40.9%)^7^, Gimbi (41.7%)^22^, Alert Hospital (41.2%)^31^, Debrebrihan referral hospital (38.9%)^23^, Addis Ababa (35.5%)^24^, Somalia (33%)^14^, Pakistan (32.2%)^8^, Jimma (30.2%)^25^, and Debremarkos town (11.7%)^26^. This might be due to differences in socioeconomic status, study period, sample size, the studied population, and data collection tools. For instance, the study conducted in China^7^ used the burn depression checklist, Western Uganda^13^ used the Center for Epidemiological Studies’ depression scale, Addis Ababa^24^ used the Beck depression inventory-II, and we used PHQ-9.

The employed patients were 78% less likely to have depression than unemployed patients. This finding was supported by a study conducted in Nigeria and Cameroon that revealed unemployment and low income were associated with depression among people living with HIV^15,32^. This might be because being employed will reduce the socioeconomic burden that might impose additional stress on HIV-positive patients. This double burden of stress might lead the patients to depressive disorder.

HIV-positive patients with the most recent CD4 count ≤ 200 cells/mm^3^ were seven times more likely to have depression than patients with CD4 count ≥ 500 cells/mm^3^. This finding was consistent with a study conducted in a tertiary hospital in South Western Nigeria, Cameroon and by the centers for AIDS research^15,32,33^. This might be because low CD4 count might be associated with opportunistic infections, which further bring additional worries, stress and physical disabilities.

Patients taking ART for ≤ 24 months were five times more likely to have depression than their counterparts. This finding was in line with a study conducted in rural Uganda and Spanish^34,35^. This might be attributed to ongoing counseling and support the patients might receive about HIV diagnosis and treatment, which might reduce mental stress and anxiety leading to depressive symptoms.

HIV-positive patients with at least one co-morbid chronic non-communicable disease (diabetes, hypertension, cardiac diseases and asthma) were eight times more likely to have depression than their counterparts. This finding was supported by a study conducted by Watkins CC and Treisman GJ in Johns Hopkins Hospital, which found neuropsychiatric symptoms, including depression, cognitive impairment, and substance abuse, are common among HIV-infected patients with chronic co-morbid conditions^36^. This might be due to co-morbid chronic non-communicable diseases, which will add tremendous pressure to the existing challenge in the fight against HIV.

## Limitations of the study

The study’s cross-sectional design makes it impossible to determine the temporal link between various factors and depression. Because the study was conducted in a hospital, the results may not apply or generalize to the entire population. We performed quantitative research to assess factors associated with depression among HIV patients. If qualitative methods, such as focus groups and in-depth interviews, had been combined with this quantitative study, more information concerning depression among HIV-positive patients would have been identified. Furthermore, hormonal tests like thyroid function tests, serum cortisol, testosterone, estrogen, and progesterone levels, all of which might be associated with depression, were not available in this hospital during the study.

## Conclusion

A significant proportion, 52.4% of HIV-positive patients taking ART, had depression. Patient employment status, most recent CD4 count, months on ART, and chronic non-communicable diseases were factors associated with depression among HIV-positive patients. Employed patients were less likely to have depression. However, patients with most CD4 counts of less than 200 cells/mm^3^ and those who took ART for ≤ 24 months and had chronic non-communicable diseases were at increased risk of developing depression.

## Recommendation

The Ministry of Health and partners working on HIV need to strengthen mental health screening and treat depression among PLWHA with due attention on unemployed patients, low CD4 count, patients newly initiated on ART and with co-morbid chronic non-communicable patients. Further longitudinal research on risk factors of depression should be conducted to strengthen and broaden the current findings.

## Data Availability

The dataset analyzed during the current study is available from the corresponding author upon reasonable request.

## Abbreviations

AIDS: Acquired immune deficiency syndrome
ART: Antiretroviral therapy
BMI: Body Mass Index
BSC: Balanced score card
CD4: Cluster of differentiation
HIV: Human immune virus
PHQ: Patient health questionnaire
PLWHA: People living with HIV/AIDS
WHO: World Health Organization
